# Community Trait Distributions Drive Biomass Stand Allocation Trade‐Offs in Karst Forests

**DOI:** 10.1002/ece3.72491

**Published:** 2026-02-08

**Authors:** Dong‐Mei Yuan, Ling‐Bin Yan, Feng Liu, Hui‐Min Zhang, Xiu‐Gang Cao, Yuan Liu, Zhi‐Fei Chen, Li‐Fei Yu

**Affiliations:** ^1^ Key Laboratory of Plant Resource Conservation and Germplasm Innovation in Mountainous Region (Ministry of Education), College of Life Sciences/Institute of Agro‐Bioengineering Guizhou University Guiyang China; ^2^ Research Center of Karst Ecology and Environment Guizhou University Guiyang China

**Keywords:** biomass, climax community, karst, trade‐offs, trait‐driven theory

## Abstract

Community biomass allocation is jointly determined by habitat conditions and plant functional traits. Studies of biomass allocation patterns in topographic—soil climax communities of karst ecosystems remain scarce. According to the trait‐driven paradigm, topographic gradients and soil properties indirectly influence karst forest biomass, via their control over community—level functional structure. In the 25—ha Maolan Dynamic Plot of the Karst Forest Ecosystem in South China, we compiled 1255 high—quality trait records for six key plant functional traits related to biomass from 48 dominant species, individual biomass data for 12,354 stems, and fine‐scale environmental variables. Partial least—squares structural equation modeling (PLS—SEM) was used to quantify the direct and indirect factors affecting biomass allocation in this climax karst forest community. We observed that the trade‐offs in biomass among different forest layers were more effective in predicting the biomass status of natural communities (*R* ^2^ = 0.69). Topographic heterogeneity acted as an environmental filter, driving the assembly of distinct karst climax communities. Community—level trait distributions and abiotic variables significantly influenced both community biomass and its trade‐offs, although trait patterns explained biomass trade‐offs more effectively than environmental factors. PLS—SEM identified slope position as the primary driver of biomass trade‐offs in the karst climax communities, with community—level variation in specific leaf area (SLA) mediating biomass allocation. Slope position decline reduced the community—weighted mean of functional traits (SLA, Wood density, Leaf nitrogen content) and concurrently increased biomass of the stable layer. In parallel, lower community—weighted variance of traits (SLA) attenuated biomass loss in the regeneration layer. These results underscore the pivotal role of trait composition in mediating biomass partitioning at the community scale.

## Graphical abstract

In this study, multi‐source data (functional traits of dominant species, topography‐soil parameters, and biomass) of the dynamic monitoring plot of Maolan Dynamic Plot of Karst Forest Ecosystem in South China were collected. The partial least squares structural equation model (PLS‐SEM) was adopted to study the biomass trade‐off strategy of the karst forest community at the “topography‐soil” extremum. It is revealed for the first time that: (1) The forest layer distribution model of community biomass has the advantage of biomass trade‐off in the karst polar community. (2) The karst climax community is jointly influenced by the “topographic – soil” environment, and the community functional structure shows obvious differences. (3) Based on the theory of trait drive, break through the cognitive framework of the functional structure of the community affected by environmental factors from the weighted moments of functional trait communities. This research aims to deepen the understanding of the construction mechanism of non‐zonal vegetation communities in the study of subtropical karst forest ecosystems.

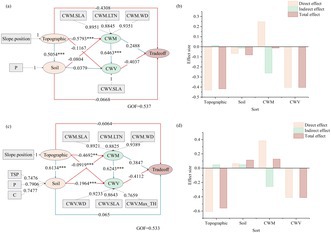

## 1 Introduction

Biomass allocation strategy denotes the adaptive partitioning of finite assimilated biomass among organs and functional guilds throughout plant ontogeny (Wang et al. 2024). The existing biomass provides the material and energy for the growth of plants, thereby restricting their growth dynamics. Biomass allocation strategies reflect critical trade‐offs in resource acquisition, storage, and partitioning within plant communities, with these strategies being shaped by both the community composition and environmental constraints (Younginger et al. 2017; An et al. 2025). Plant functional traits serve as robust indicators of community structural organization and provide insights into community‐level responses to environmental gradients. Consequently, the analysis of traits has emerged as a fundamental framework in functional ecology (Liu et al. 2020). Extensive empirical evidence has demonstrated that community‐level functional trait structure—encompassing functional parameters, diversity, and redundancy—exhibits stronger predictive power for ecosystem productivity compared to traditional species diversity metrics (Violle et al. 2007). The trait‐driven theory posits that shifts in the relative importance of assembly processes—including abiotic filtering, biotic interactions, and stochastic (neutral) processes—govern the distributional properties of community functional traits (e.g., mean, variance, skewness, and kurtosis). These trait distribution dynamics, in turn, impact plant community functioning (Denelle et al. 2019; Yao et al. 2023). Liu, Sack, et al. (2022) demonstrated that plant stomatal traits exhibited adaptive convergence under drought stress, with community‐level stomatal trait distributions explaining 68% of the variation in ecosystem productivity. This finding highlights the critical role of stomatal optimization in maintaining photosynthetic efficiency under water‐limited conditions. The mass ratio hypothesis holds that ecosystem processes are predominantly controlled by the functional traits of dominant species, with community‐level biomass allocation patterns being directly determined by their trait values (Grime 1998). These allocation trade‐offs can be quantitatively characterized through the community‐weighted mean (CWM) of relevant functional traits (Tortorelli et al. 2022). Functional trait diversity captures interspecific niche differentiation in resource utilization and biomass allocation through complementary effects, with community‐weighted variance (CWV) of traits quantitatively reflecting the dispersion of functional strategies within plant communities (García‐Palacios et al. 2018). Additionally, the community‐weighted skewness (CWS) and kurtosis (CWK) of functional traits quantify functional rarity and uniformity within plant communities, reflecting the integrated effects of biotic/abiotic filtering and dispersal limitation on community assembly (Enquist et al. 2015). Understanding how plant functional traits mediate community biomass allocation trade‐offs is crucial for developing science‐based forest management and ecological restoration strategies.

Environmental constraints, particularly hydrological regimes and topographic gradients, exert threshold‐mediated regulation on the patterns of community biomass allocation (Pierick et al. 2023). For example, as the forest‐shrub‐grassland ecosystem develops, the proportion of underground biomass gradually increases (Ma et al. 2021). The direct environmental control over plant carbon allocation favors biomass investment toward tissue expansion (sink enhancement) rather than photosynthetic capacity (source development), reflecting an adaptive strategy for structural growth under resource constraints (Fatichi et al. 2014). The distribution of plant community biomass is strongly mediated by local soil conditions. Soil physicochemical properties directly govern biomass allocation patterns through water and nutrient availability, while simultaneously driving adaptive optimization in heterogeneous environments by modulating key functional traits, including the root: shoot ratio and the leaf mass fraction (Wang et al. 2023). This observation aligns with Optimal Partitioning Theory (OPT), which posits that adaptive biomass allocation enhances resource‐use efficiency, facilitates organic matter sequestration, and sustains community stability through optimized functional trade‐offs (Thornley 1972; Kobe et al. 2010; Craven et al. 2018). The environment is an important control point for understanding the relationship between biomass trade‐offs. Thus, the assessment of abiotic factors is necessary for analyzing the community trait attributes and trade‐off relationships that control forest biomass (Zhang et al. 2022).

A climax community represents the terminal successional stage in ecosystem development and is characterized by a self‐sustaining equilibrium, where the species composition demonstrates optimal adaptation to prevailing environmental conditions through long‐term ecological processes (Whittaker 1953). Karst climax forest communities, as archetypal topographic–soil climax vegetation, exhibit strong dependence on localized environmental controls (Jiang et al. 2014). The complexity of the terrain supports diverse karst microhabitats, and the plant assemblages in these habitats have unique functional traits that affect the community structure (Frei et al. 2025). At the community level, plant functional traits characterized by lower specific leaf area (SLA) and higher leaf dry matter content (LDMC) reflect an adaptive conservative resource‐use strategy (Tang et al. 2016; Liu, Hu, et al. 2022). During late‐successional stages, functional traits exhibit heightened contributions to the dimensionality of community functional space associated with enhanced resource‐use efficiency (Li et al. 2025). In the karst climax community stage, there is significant interspecific variation in plant functional traits (Tang et al. 2016), facilitating the formation of mature forests with diverse functional strategies (Zhang et al. 2013; Shui et al. 2022; Meng et al. 2024). Topographic factors and soil nutrient availability may indirectly regulate karst forest productivity by mediating shifts in the functional structure of the plant community (Hu et al. 2017; Zhang et al. 2022). Nevertheless, the mechanistic links between key environmental drivers (e.g., topographic and soil factors) and the biomass allocation strategies of climax communities in karst ecosystems remain poorly understood.

Although environmental filters and functional traits collectively govern plant biomass partitioning, the magnitude of their respective effects remains to be systematically evaluated. In mature karst climax communities, we hypothesize that topographic and soil factors will jointly regulate the community distribution of plant functional traits, and that this in turn will mediate the variation in community‐level biomass. Here, we tested the power of trait‐driven theory to explain community ecological strategies (i.e., biomass trade‐offs). We sampled karst climax communities within the Maolan Dynamic Plot of the Karst Forest Ecosystem in South China. We used multiple linear regression with partial least squares structural equation modeling (PLS‐SEM) to integrate key soil, topographic, and trait factors that were correlated with biomass. We addressed the following issues: (1) Are the effects of functional traits in karst communities on community production strategies (biomass trade‐offs) consistent with trait‐driven theory? (2) How do community trait distributions explain biomass trade‐off relationships in karst forests?

## 2 Materials and Methods

### 2.1 Study Sites

The Maolan National Nature Reserve (25°09′20″–25°20′50″ N, 107°52′10″–108°45′40″ E) in Libo County, Qiannan Bouyei‐Miao Autonomous Prefecture of Guizhou Province, represents a well‐preserved karst climax vegetation community. Situated in the transitional zone between the southern Guizhou Plateau and the Guangxi hilly plains, the reserve features a characteristic karst landscape with northwest‐to‐southeast topographic relief. The lithology consists primarily of pure limestone and dolomite, with black calcareous soil as the dominant pedogenic type. Karst landscapes are characterized by high proportions of exposed bedrock, a discontinuous soil distribution, shallow soil layers, and significant habitat fragmentation, resulting in diverse microhabitats that include soil surfaces, rock gullies, bare rock surfaces, and rock crevices. The study area exhibits a typical humid subtropical monsoon climate, with a mean annual temperature of 15°C, annual precipitation of 1320.5 mm, mean annual relative humidity of 83%, and approximately 87% forest coverage. The vegetation types in reserve are primarily non‐zonal evergreen deciduous broad‐leaved forests in the mid‐subtropical zone, and the dominant species in the tree layer are *Platycarya strobilacea* , *Boniodendron minus*, *Lithocarpus glaber*, and *Clausena dunniana*. The dominant species in the shrub layer are *Lindera communis*, *Cornus wilsoniana*, *Viburnum henryi*, and *Mahonia fortunei*. The dominant species in the herb layer are *Selaginella uncinata* , *Pilea semisessilis*, *Asarum heterotropoides*, and *Hedera nepalensis*.

### 2.2 Sampling Points and Data Collection

**FIGURE 1:**
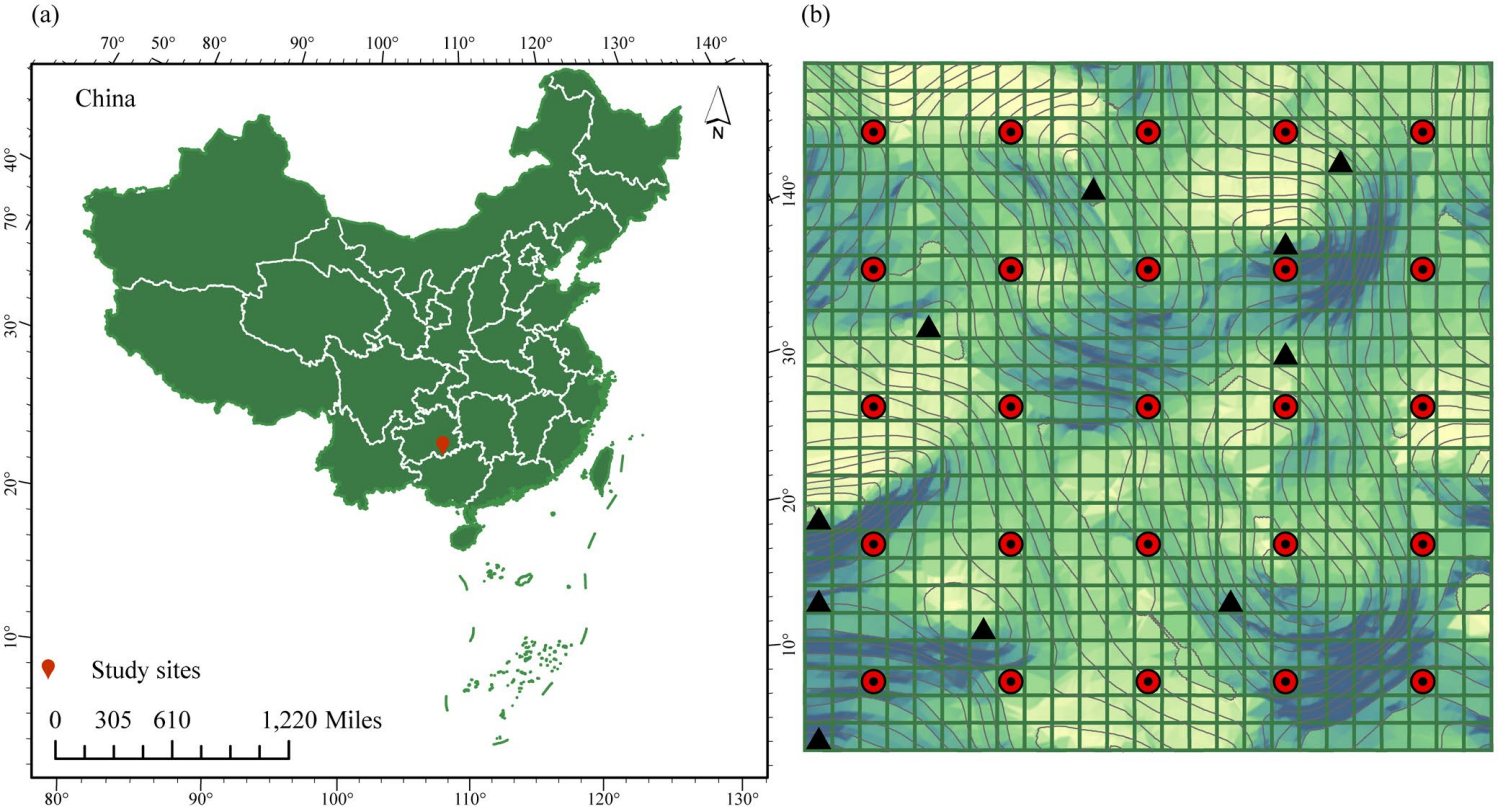
Sample Layout Map. (a) Location of the research area. Each cell in (b) represents a 20 m × 20 m quadrat, and the red circle and black dots are marked as systematic sampling quadrats. After analyzing the topographic characteristics of the systematically sampled plots, supplementary quadrats were established according to the representativeness principle. The quadrats (marked with black triangles in b) were positioned so that all sampling quadrats roughly covered all topographic factor categories present in the large plots.

The study area was located in the Maolan Dynamic Plot of the Karst Forest Ecosystem in South China, established in 2022 (Figure 1). The plot covers an area of 25 hm^2^. There is a small degree of human disturbance. The plot features an original karst evergreen, deciduous broad‐leaved mixed forest located between the buffer zone and the experimental part of the protected area.

### 2.3 Setting Quadrats

From July to August 2023, based on systematic field investigation of landform, vegetation type, community composition and distribution, and other factors in Maolan large sample plots, small sample plots were selected by using systematic sampling schemes to ensure uniformity. The sampling squares were chosen at 80‐m intervals. A total of 35 small quadrants were selected for sampling.

### 2.4 Plant Community Survey

We surveyed all woody plants with a diameter at breast height (DBH) ≥ 1 cm, recording species identity, tree height, and DBH for each individual. The census documented 12,354 woody plants comprising 310 species from 163 genera and 74 families. Using hierarchical clustering with Ward's minimum variance method, we classified the Maolan Karst climax forest communities into four distinct associations: (1) Ass. *Lindera communis*, (2) Ass. *Lindera communis + Lithocarpus henryi*, (3) Ass. *Carpinus pubescens +* *Platycarya strobilacea* *+ Acer sycopseoides*, (4) Ass. *Platycarya strobilacea* .

### 2.5 Environmental Data

#### 2.5.1 Terrain Data Acquisition

A Digital Elevation Model (DEM) of the Maolan study site was utilized to extract topographic parameters including aspect, altitude, and slope. Rock exposure was quantified through field measurements within each quadrat. Slope position was categorized into upper, middle, and lower sections based on the quadrate's relative elevation along the mountain slope.

#### 2.5.2 Soil Data Acquisition

Random sampling was carried out for each quadrate. After removing debris such as dead leaves and stones on the surface of the sampling point, 500 g of soil sample was collected from the soil surface (0‐10 cm soil layer), and a soil ring knife was collected. Three replicates were set for each quadrat. The soil physical properties and water retention characteristics were determined using a soil ring knife. Following sample collection, 500 g of soil was transported to the laboratory for air‐drying and sieving (2 mm mesh) as pretreatment prior to nutrient analysis. Soil nutrient indices were determined according to the standardized methods described in Bao Shidan's “Soil Agrochemical Analysis.” The contents of carbon, nitrogen, and phosphorus were quantified through elemental analyses. Soil pH was measured potentiometrically using a calibrated pH meter with glass electrodes. Soil factor indicators are shown in Table 1.

**TABLE 1:** Soil index.

Category	Divisor	Unit
Soil nutrient content	Soil carbon content, C	g·kg^−1^
	Soil nitrogen content, N	g·kg^−1^
	Soil phosphorus content, P	g·kg^−1^
Soil physical characteristics	Soil bulk density, SD	g·cm^−3^
	Total soil porosity, TSP	%
	Soil capillary porosity, SCP	%
	Soil pH	—
Soil water retention characteristics	The natural moisture content of the soil, NMCS	%
	Soil saturated water‐holding capacity, SWHC	%
	Soil capillary water retention, SCWR	%
	Soil non‐capillary water retention, SNCR	%
	Soil mass water content, SMWC	g·kg^−1^
	Soil volumetric water content, SVWC	g·L^−1^
	Soil water storage, SWS	mm
	Soil saturated water capacity, SSWC	mm
	Soil capillary water capacity, SCWC	mm
	Soil non‐capillary water capacity, SNCWC	mm

### 2.6 Quantification of Plant Functional Traits

We quantified six key plant functional traits linked to biomass, including Maximum plant height (Max_TH), the Maximum DBH of a plant (Max_DBH), Wood density (WD), Leaf nitrogen content (LTN), Specific leaf area (SLA), and Leaf dry matter content (LDMC). Plant functional traits were measured following the Manual of Standardized Measurement of Global Plant Functional Traits (Pérez‐Harguindeguy et al. 2013). During the plant growth season (July–August 2023), we collected 1255 plant samples from mature individuals of the dominant species across 35 sampling quadrats. Since the dominant species in different plots were not the same, we calculated the dominant species in 35 plots, totaling 48 plants (Table S4). Community‐weighted moments of functional traits—including the community‐weighted means (CWM), community‐weighted variance (CWV), community‐weighted skewness (CWS), and community‐weighted kurtosis (CWK)—were calculated as follows (Brun et al. 2022).

(1) $$\text{CWM}=\sum_{i}^{n}w_{i}x_{i}$$

(2) $$\text{CWV}=\sum_{i}^{n}w_{i}{\left(x_{i}-\mathrm{CWM}\right)}^{2}$$

(3) $$\text{CWS}=\sum_{i}^{n}w_{i}\frac{{\left(x_{i}-\mathrm{CWM}\right)}^{3}}{{\text{CWV}}^{3/2}}$$

(4) $$\text{CWK}=\sum_{i}^{n}w_{i}\frac{{\left(x_{i}-\mathrm{CWM}\right)}^{4}}{{\text{CWV}}^{2}}$$

In the formulas, *n* denotes the total number of species within the community, $x_{i}$ corresponds to the mean value of specific functional traits of species i, and $w_{i}$ indicates the relative biomass contribution of species i to the total community biomass.

In this study, we focused on estimating the aboveground biomass. For woody plants within the quadrats, biomass was calculated using species‐specific allometric equations developed for karst‐region tree species, with the following formula:

(5) $$W=0.0414{\left(D^{2}H\right)}^{0.9534}$$

In the formula, *D* is the diameter at breast height and *H* is the height of the tree. A total of 12,354 individual biomass values were calculated in this study.

### 2.7 Biomass Allocation Model

To understand the optimality of the biomass allocation model of karst climax communities, we attempted the predictive ability of multiple allocation models for the biomass of karst climax communities. Biomass trade‐offs allocation models include: (1) The stratified distribution was allocated according to the forest layer where the trees developed, primarily including the regeneration layer (Regeneration, DBH = 1 cm), the succession layer (Succession, 1 cm < DBH < 5 cm), and the Stable layer (Stable, DBH ≥ 5 cm). (2) Component type distribution, allocated according to plant growth morphology, including trunk growth type and branch growth type. (3) Mycorrhiza type distribution, where biomass is allocated according to the symbiosis between mycorrhiza and plants, including Ericoid mycorrhiza (ERM), Azorhizoboium, Ectomycorrhiza, and Azorhizoboium (ECM), Endophyte, and Arbuscular Mycorrhiza (AM). (4) A growth type distribution, where different groups can be assigned according to the visible structures of the plants, including shrubs, shrubs or small trees, woody vines, and trees. (5) Phenological characteristics of leaves, where groups are assigned according to the phenological characteristics (or leaf lifespan) of leaves, leaf habits can be classified into three types: evergreen, semi‐evergreen and deciduous trees. To evaluate the performance of different biomass allocation models in predicting community biomass within karst forest climax communities, we employed the root mean square error (RMSE) as a quantitative metric.

(6) $$\text{RMSE}=\sqrt{\frac{1}{n}\sum_{i=1}^{n}{\left(\hat{y_{i}}-y_{i}\right)}^{2}}$$

In the equation, $y_{i}$ represents the observed total community biomass, $\hat{y_{i}}$ denotes the predicted total community biomass from the model, and n is the number of sampling plots. We developed a linear regression model using biomass data from 25 plots as the training dataset to predict total community biomass across different allocation strategies. The biomass from 10 plots was used as the test set. The established model generated predicted values, and the RMSE between predicted and observed measured values was computed as the trade‐off metric. A small RMSE value indicates better model performance, demonstrating closer agreement between the predicted and observed community biomass. This approach enables quantitative comparison of different biomass allocation models to determine their relative performance.

We collected data on the growth patterns and leaf habits of dominant species from the iPlant Plant Intelligence—Plant Species Information System (https://www.iplant.cn/). Information regarding the mycorrhizal status of dominant species was obtained from the FungalRoot Database (https://www.gbif.org/dataset/744edc21‐8dd2‐474e‐8a0b‐b8c3d56a3c2d), with additional mycorrhizal data referenced from the relevant literature (Soudzilovskaia et al. 2020).

### 2.8 Statistical Analysis

In this study, the optimal model for the karst forest climax community was stratified biomass allocation. The biomass ratio between forest layers reflects the trade‐offs in biomass distribution among different layers. Data preprocessing was performed with the “Hmisc” and “dplyr” packages in R 4.4.2. RS1 represents the trade‐off relationship between regeneration and successional biomass, RS2 denotes the trade‐offs relationship between regeneration and stable biomass, and SS indicates the trade‐offs relationship between successional and stable biomass. Spearman correlation analysis was used to assess the relationship between biomass and various forest layers. Additionally, linear regression was conducted to examine the impacts of biomass in different forest layers and their trade‐offs on the overall community biomass. An initial Spearman correlation analysis was conducted to investigate the impacts of environmental factors on the trade‐off relationships of karst forest biomass. Considering the unique characteristics of the karst environment and its ecological functions, we removed one factor from each correlated pair with a correlation coefficient ≥ 0.8 to avoid multicollinearity (Figure S1). Spearman correlation analysis was performed using the “Hmisc” package in R 4.4.2. Linear regression was conducted with the “car” and “MASS” packages in R 4.4.2. The retained abiotic factors encompassed topographic aspects (slope position, slope, aspect, and rock exposure) and soil attributes (nutrients: C, P; physical characteristics: SD, TSP, SCP, and SNP; water holding capacity: NMCS, SWHC, SNCR, SVWC, and SWS). Principal component analysis (PCA) was conducted on the filtered abiotic environmental factors. The PCA results were utilized to identify the ecological characteristics and distribution preferences of karst climax forest communities. Multiple regression and variance decomposition methods were employed to identify significant predictors of biomass trade‐offs, in conjunction with the results of the Spearman correlation analysis, to elucidate the mechanisms underlying the effects of abiotic environment and community‐weighted moments on biomass trade‐offs. The PCA was performed using the “vegan” and “FactoMineR” packages in R 4.4.2.

PCA was performed on the community distribution characteristic values of all plant functional traits (Figure S3). The first three axes explained more than 90% of the variation and were selected to determine the main community character distribution feature factors. Based on the results of the multiple regression analysis and variance decomposition, the community traits significantly influencing biomass trade‐offs were selected for Partial Least Squares Structural Equation Modeling (PLS‐SEM). The same method was applied for screening environmental factors. The multiple regression and variance decomposition were conducted using the “relaimpo” and “stats” packages in R 4.4.2. The retained significant factors are listed in Table 2.

**TABLE 2:** Final screening factors.

Trade‐offs layer	Community traits	Environmental factor
RS2	CWM.WD, CWM.LTN, CWV.Max_TH, CWV.LDMC, CWM.SLA, CWV.SLA	Slope.position, P
SS	CWM.WD, CWV.Max_TH, CWM.LTN, CWV.WD, CWM.SLA, CWV.SLA	Slope.position, P, C, TSP

PLS‐SEM was used to analyze the factors listed in Table 2, and to quantify the effects of different variables on the trade‐offs in biomass allocation. The results were used to explore the combined effects of the abiotic environment and community character distribution on biomass trade‐offs. The influence path modeling based on PLS‐SEM was accomplished through the “lavaan” and “plspm” packages in R 4.4.2. PLS‐SEM used the Goodness‐of‐Fit (GOF) index to test the quality of simulation results. It is generally accepted that the model with GOF > 0.7 has strong predictive ability and a good fitting effect. Values in the range 0.3 ≤ GOF ≤ 0.7 indicate that the model's fit is medium, and it has particular explanatory power. A GOF < 0.3 indicates poor model fitting. Finally, Variance Partitioning Analysis (VPA) and hierarchical segmentation were employed to identify the primary factors influencing the relationship between biomass trade‐offs.

All images were visualized using the “ggplot 2” package in R 4.4.2 and optimized by Adobe Illustrator 2019. The map of the study area was drawn using ArcGIS Pro 3.0.1.

## 3 Results

### 3.1 Biomass Allocation Trade‐Offs Characteristics

In the biomass allocation model of karst forest climax communities (Table S1), leaf habit distribution patterns (semi‐evergreen/evergreen/deciduous *p* = 0.048, semi‐evergreen/deciduous *p* = 0.013), growth form distribution (woody vines/trees *p* = 0.031), and vertical stratified patterns (regeneration/succession and regeneration/succession/stable *p <* 0.000) significantly predicted community biomass. Among the models, the stratified biomass allocation approach demonstrated the strongest predictive power (*R* ^2^ = 0.69, RMSE = 1.6).

**FIGURE 2:**
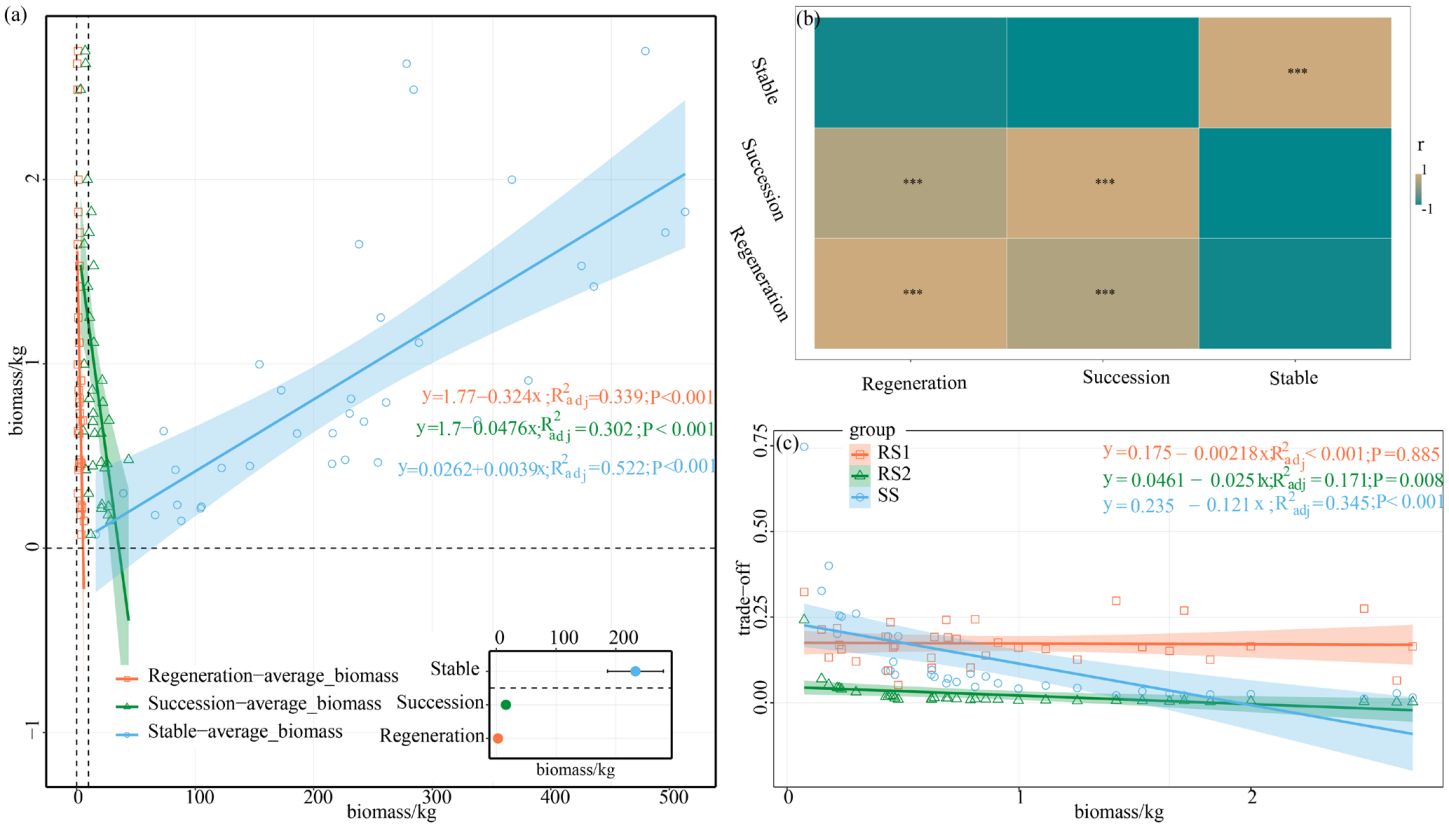
Relationship between biomass allocation patterns and total community biomass across forest strata. (a) Contribution of stratified biomass (regeneration layer, succession layer, and stable layer) to total community biomass, with the inset boxplot (lower right) showing the mean biomass distribution across strata. (b) Correlative relationships between biomass allocations in different forest strata. (c) The effects of interstrata biomass trade‐offs on total community biomass.

The biomass of the regeneration, succession, and stable layers significantly influenced community‐level biomass (Figure 2). There was a significant negative correlation between community biomass and both regeneration and succession layer biomass, whereas the stable layer biomass showed a significant positive correlation with community biomass (Figure 2a). Furthermore, the regeneration layer biomass was significantly positively correlated with succession layer biomass; however, there was no significant negative correlation was found between the stable layer biomass and either the regeneration or succession layer biomass (Figure 2b).

The biomass trade‐offs among forest layers differentially influenced the total community biomass (Figure 2c). The biomass trade‐offs of RS2 and SS had a significant impact on community biomass. The community biomass decreased with the increase in the degree of biomass trade‐offs. The SS biomass trade‐offs degree (*R* ^2^ = 0.345) had a more significant effect on community biomass. This indicates that prioritizing the allocation of biomass to the renewal layer or succession layer would reduce the biomass of the entire community, which may be due to the increased niche complementarity among different forest layers.

### 3.2 Influence of Abiotic Factors on Biomass Allocation Trade‐Offs Across Vertical Forest Layers

**FIGURE 3:**
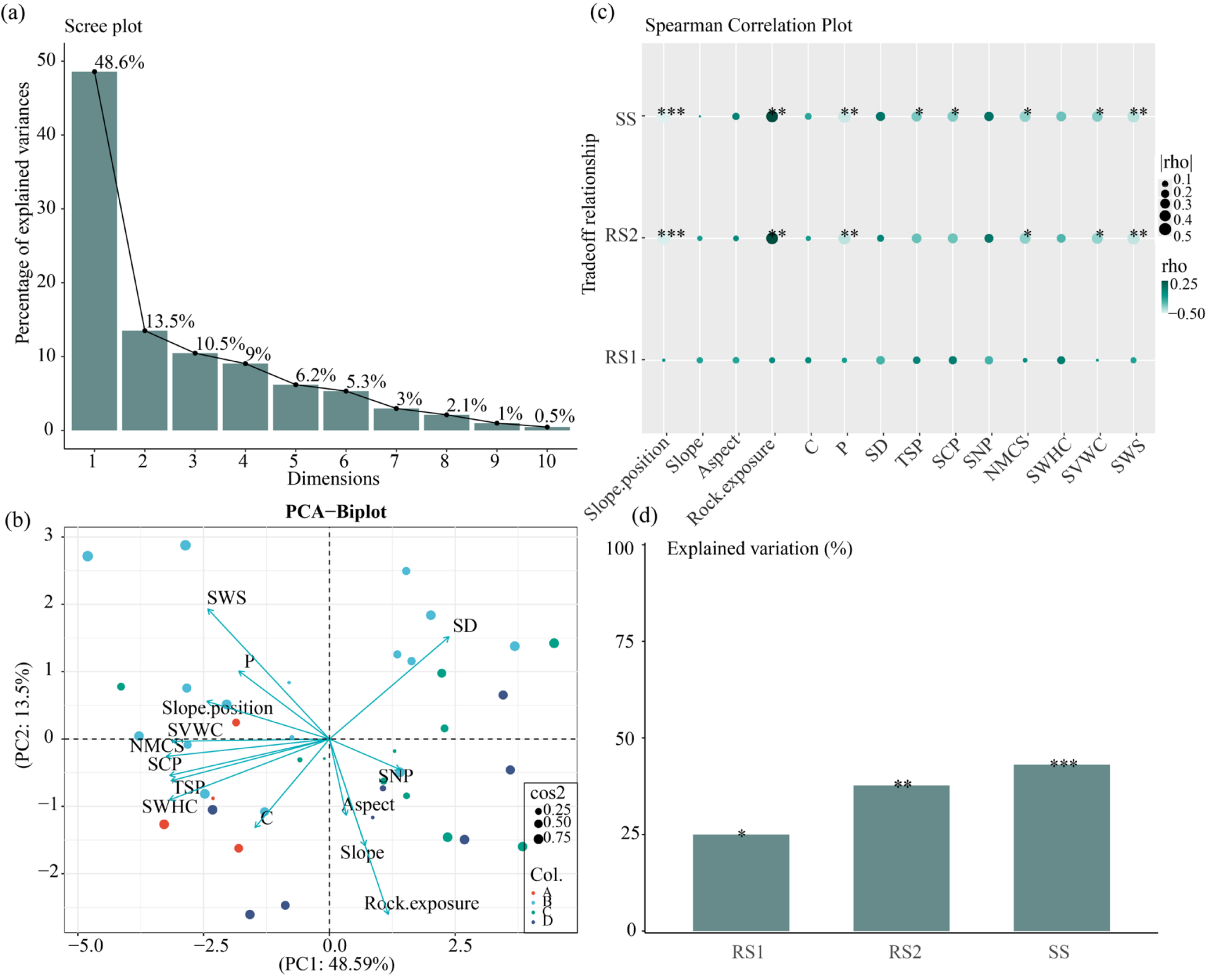
Effects of abiotic environmental factors on biomass allocation trade‐offs. (a) Variance explained by principal components (PCs) and (b) principal component analysis (PCA) biplot showing community differentiation. Point color represents distinct plant communities (A: Ass. *Lindera communis*; B: Ass. *Lindera communis* + *Lithocarpus henryi*; C: Ass. *Carpinus pubescens +* *Platycarya strobilacea* *+ Acer sycopseoides*; D: Ass. *Platycarya strobilacea* .), while point size reflects the sample importance (cos^2^ size) in the PCA space. (c) Spearman correlation matrix between environmental variables and biomass trade‐offs. (d) The variance explained (*R* ^2^) by the multiple regression model. The trade‐off value as the response variable and all environmental factors are explanatory variables. Significance levels: *p < 0.05, **p < 0.01, ***p < 0.001.

Environmental variation in the karst forest climax community was primarily driven by differences in small topography, soil nutrient content, and soil water retention performance and had a significant impact on the biomass trade‐offs between forest layers (Figure 3). The first two principal components (PC1 and PC2) collectively explained 62.09% of the total variance, primarily reflecting the variation in soil properties (Figure 3a,b). On the PC1 axis, soil factors contributed 43.07% of the variance (SD 27.05%, SNP 16.03%), while topographic factors explained 25.22% (Rock.exposure 13.30%, Slope 8.10%, Aspect 3.82%). These combined effects drove the observed variance patterns along PC1. Soil water holding properties accounted for 41.72% of the variance along PC2, followed by soil phosphorus content (21.81%) and slope position (12.10%). Distinct clustering patterns were observed among different forest community types (Figure 3b). The plant associations *Carpinus pubescens +* *Platycarya strobilacea* *+ Acer sycopseoides* and *Platycarya strobilacea* predominantly colonize rocky, high‐exposure habitats, whereas *Lindera communis* + *Lithocarpus henryi* and *Lindera communis* exhibit a preference for soils with higher nutrient availability and improved water‐holding capacity.

Environmental factors exerted significant effects on biomass allocation (Figure 3d), accounting for 35.99%, 48.69%, and 53.14% of the variation in RS1, RS2, and SS trade‐off relationships, respectively. Notably, the magnitude of these effects was strongest in the stable layer. Spearman correlation analysis demonstrated that rock exposure positively correlated with RS2 and SS trade‐off values (*p* < 0.05), whereas soil available phosphorus content and soil water‐holding capacity showed significant negative correlations (Figure 3c). These results indicate that the higher rock exposure promoted biomass investment in the regeneration and succession layers, while superior soil nutrient availability and water retention capacity favored the allocation of biomass to the stable layer.

### 3.3 Effects of the Distribution of Community Traits on Biomass Trade‐Offs Between Different Forest Layers

**FIGURE 4:**
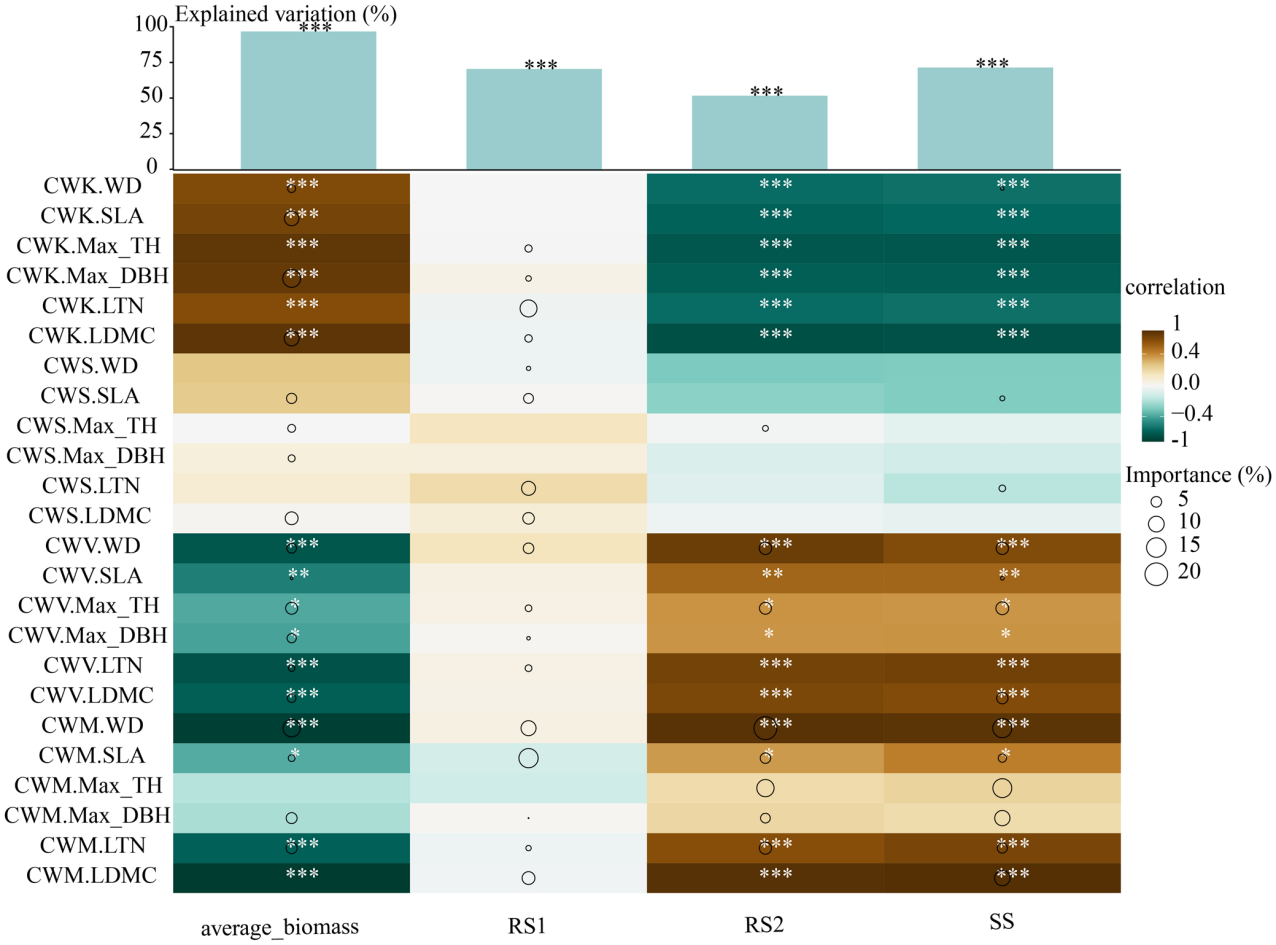
Influence of the community distribution characteristics of six functional traits on biomass and its allocation trade‐offs. The upper panel displays a bar chart quantifying the explanatory power (*R* ^2^ values) of multiple regression analyses, with trait community distribution metrics as independent variables and either total biomass or trade‐off indices as dependent variables. The lower panel presents a heatmap visualizing correlations between trait distribution characteristics and biomass allocation parameters. Circle sizes within the heatmap represent the relative importance of each trait distribution characteristic in explaining the variation in the corresponding dependent variable. The circle size represents the relative importance of each variable. Significance levels are denoted as: *p ≤ 0.05, **p ≤ 0.01, and ***p ≤ 0.001. All abbreviations are defined in Table S3.

Multivariate linear regression analyses revealed that the distribution characteristics of the six functional traits had significant effects on both total community biomass (*R* ^2^ = 95.52%) and biomass allocation trade‐offs ($R_{\mathrm{RS}1}^{2}$ = 68.35%, $R_{\mathrm{RS}2}^{2}$ = 53.91%, $R_{\mathrm{SS}}^{2}$ = 71.78%), and the main influencing factors varied (Figure 4, Table S2). The degree of RS1 trade‐offs showed a predominantly negative correlation with CWM.SLA (14.78%), while the overall CWS of the six functional traits accounted for 27.53% of the observed variation. For the RS2 trade‐off, CWM.WD (19.25%) and CWM.MAX_TH (11.67%) emerged as the primary determinants, with the overall CWM of the six functional traits collectively explaining 47.12% of the variance. The SS trade‐offs exhibited positive associations with CWM.WD (13.76%) and CWM.Max_TH (13.39%), but were negatively influenced by CWK.LDMC (7.64%). On the whole, CWM and CWV of community traits of the six functional traits were significantly negatively correlated with community biomass and significantly positively correlated with RS2 and SS trade‐offs. Conversely, the CWK of community traits of the six functional traits demonstrated inverse relationships with these parameters.

### 3.4 Potential Driving Mechanisms of Biomass Trade‐Offs

**FIGURE 5:**
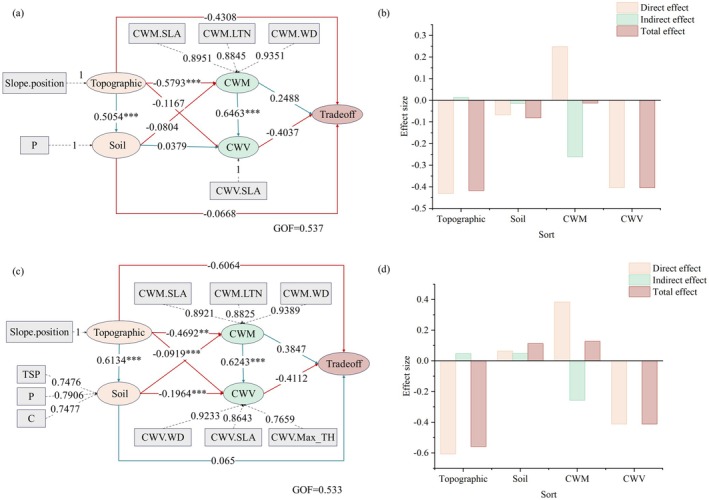
Biomass trade‐off relationships and factor effects among different forest layers. PLS‐SEM was used to analyze (a and b) the RS2 biomass trade‐offs on the model fit and (c and d) the SS biomass trade‐offs on the model fit. The line color indicates the direction of the correlation; red denotes a negative correlation, and blue indicates a positive correlation.

The PLS‐SEM analysis revealed that all examined factors exerted both direct and indirect effects on biomass trade‐offs allocation among the forest layers (Figure 4). Topographic factors, particularly slope position, demonstrated a stronger influence on biomass allocation trade‐offs than soil factors (soil C, P, and TSP) (Figure 5b,d). Notably, slope position exhibited contrasting effects, it negatively influencing the RS2 biomass allocation trade‐offs by significantly altering soil conditions (Figure 5a), while positively affecting the SS biomass allocation trade‐offs (Figure 5c). As slope position decreased, the community‐weighted mean (CWM) values of SLA, LTN and WD decreased, consequently suppressing increases in both Tradeoff_RS2_ and Tradeoff_SS_. Concurrently, the community‐weighted variance (CWV) of SLA was reduced, leading to enhanced Tradeoff_RS2_ and Tradeoff_SS_ values.

The CWM and CWV values of functional traits exhibited contrasting effects on biomass allocation patterns. The PLS‐SEM analysis of the distribution of biomass across forest layers (Figure S4) revealed that: (1) Decreasing slope position directly constrained increases in the CWM value (CWM.SLA, CWM.LTN, and CWM.WD), subsequently leading to reduced biomass in both the regeneration and succession layers while the increasing the biomass in the stable layer; (2) Soil properties exerted significant direct effects on CWV (CWV.SLA), thereby indirectly altering the distribution of biomass among forest layers, although with differential impacts. Specifically, elevated soil C and P contents and TSP were associated with significant decreases in CWV values for SLA, WD and Max_TH, resulting in increased regeneration layer biomass but decreased biomass in both the succession and stable layers.

In conclusion, our findings demonstrate that topographic factors indirectly govern the spatial distribution of plant functional traits, thereby mediating trade‐offs in biomass allocation in karst climax communities. The decline of the slope position reduced the CWM values of functional traits, leading to decreased biomass allocation in both the regeneration and succession layers, while increasing the allocation of biomass to the stable layer. Consequently, this shift suppressed Tradeoff_RS2_ and Tradeoff_SS_. Simultaneously, the decline in slope position caused the decrease in the CWV values of functional traits, but promoted the investment of biomass in the regeneration layer. This effect partially offset the CWM‐driven biomass allocation pattern, indicating a compensatory mechanism between trait mean and variance in shaping the vertical distribution of biomass. This indicates that decreases in slope position will produce a decrease in the optimal values of community functional traits, which will further promote the allocation of community biomass to the stable layer and maintain community stability by increasing individual biomass input in the later stages of community plant reproduction. Furthermore, the community‐level variation in SLA modulates biomass distribution patterns, partially compensating for CWM‐driven shifts in allocation. Together, these mechanisms maintain a balanced biomass distribution across forest strata (i.e., the regeneration, succession, and stable layers).

### 3.5 Variation in the Modules of Biomass Trade‐Offs and Their Relative Contributions

**FIGURE 6:**
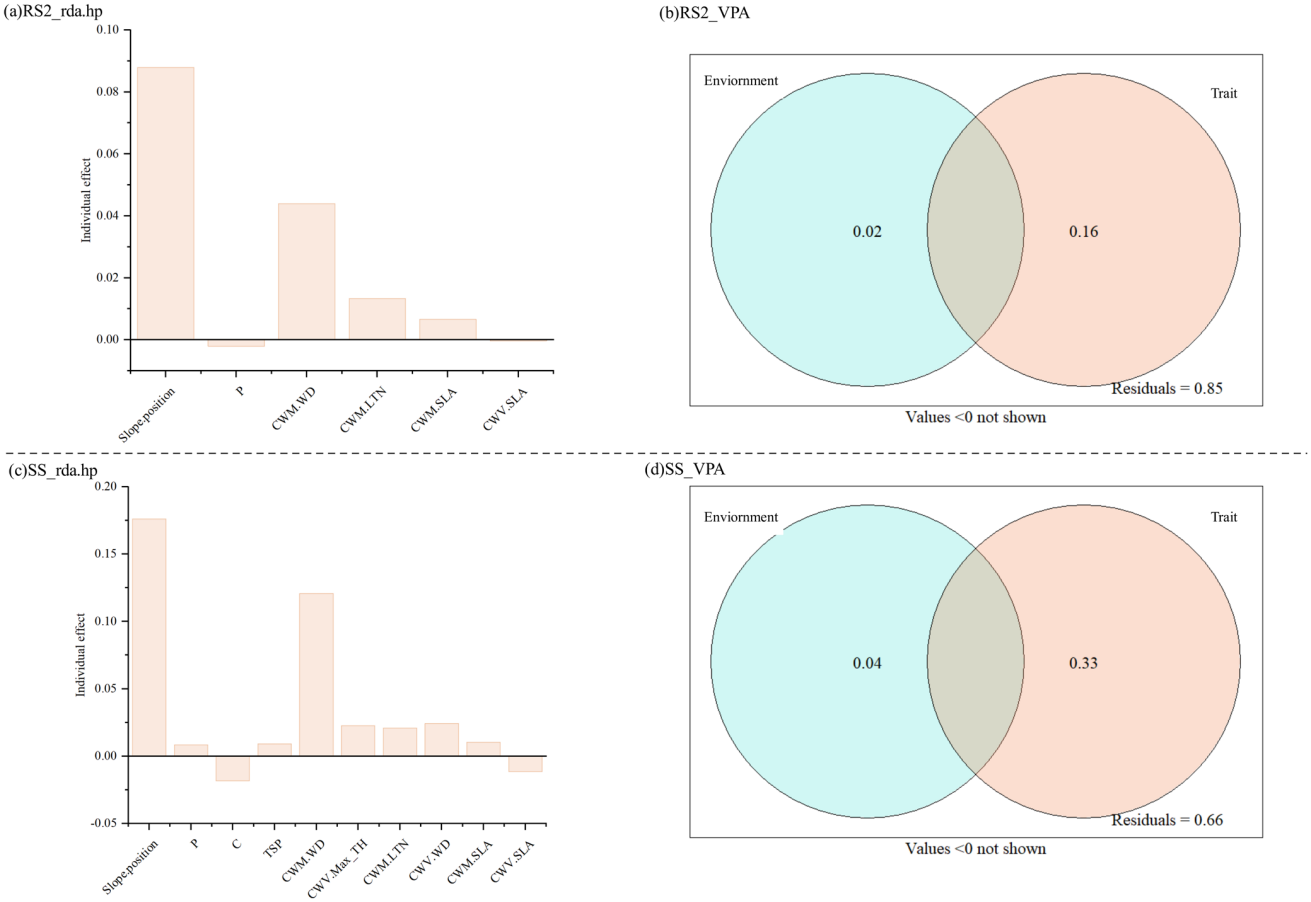
Key environmental drivers influencing biomass allocation: (a) RS2 biomass trade‐offs and (b) SS biomass trade‐offs.

Variance Partitioning Analysis revealed that the distribution of community traits explained a substantially greater proportion of biomass trade‐offs variation (16%–33%) than environmental variables (2%–4%) (Figure 6b,d). Hierarchical segmentation analysis further quantified the independent contributions of each predictor to the variation in biomass trade‐offs (Figure 6a,c). Among all variables, slope position exhibited the strongest independent effect (explaining 8.79%–17.6% of the variation), followed by CWM.WD (4.39%–12.08%). The CWM values of functional traits (CWM.SLA, CWM.LTN, and CWM.WD) accounted for 6.38% of the variation in RS2 biomass trade‐offs, while it explaining 15.2% of the variation in SS biomass trade‐offs. Additionally, the CWV of functional traits (CWV.SLA, CWV.Max_TH, and CWV.WD) contributed to 3.35% of the variation in SS biomass trade‐offs.

## 4 Discussion

### 4.1 The Forest Layer Allocation Model Best Explains in the Biomass Balance of Karst Forest Communities

Biomass allocation patterns reflect plant adaptive strategies to contrasting environments (Yang et al. 2025). Relative to traditional allocation models (Jiang and Wang 2017; Bai et al. 2025), the vertical trade‐offs among forest layers (renewable‐succession‐stable layer) more accurately predicted the biomass dynamics of the climax karst forest under field conditions (Table S1). Previous studies have primarily examined leaf economic traits and mycorrhizal associations to assess karst forest community biomass, with emphasis on photosynthetic performance and acquisition of belowground resources (Falster et al. 2011; Ma et al. 2025). In karst ecosystems, the combined effects of exposed bedrock, a discontinuous soil distribution, and nutrient depletion have substantially intensified abiotic stresses on plant communities (Guo et al. 2024). In karst climax communities, vertical stratification (i.e., distinct canopy‐understory layering) serves as the predominant adaptation mechanism to resource limitation, outperforming mycorrhizal symbiosis or leaf economic traits in optimizing light capture and nutrient partitioning. This structural differentiation directly mediates niche complementarity among co‐occurring species, ultimately enhancing community‐level fitness under abiotic stress (Laurans et al. 2014; Niklaus et al. 2017; Matsuo et al. 2024).

### 4.2 The Distribution of Community Traits Was the Main Factor Affecting the Biomass Trade‐Off Relationships

Biomass allocation trade‐offs within the climax karst forest, were jointly determined by abiotic filters and the community distribution characteristics of functional traits. Although karst‐specific environmental factors independently explained only 2%–4% of the variation in community biomass allocation trade‐offs (Figure 6), distinct climax assemblages were nevertheless clearly segregated along abiotic gradients (Figure 3b). These patterns demonstrate that local‐scale environmental heterogeneity strongly regulates the spatial organization of the karst climax community. Environmental filtering during climax community drives community differentiation, optimizing niche partitioning of light and nutrient resources to maximize adaptive allocation in these resource‐limited habitats. The trait‐gradient boundary effect (TGBE) suggests that the ranges of functional traits impose regional constraints on plant community differentiation (Denelle et al. 2019). Functional structure differences are a manifestation of community differentiation, and changes in functional traits will have varied impacts on community functions.

In our research results, the community distribution characteristics of six plant functional traits closely related to biomass all have an explanatory rate of more than 50% for the changes in community biomass and biomass allocation trade‐offs (Figure 4). Moreover, compared with environmental characteristics, it also has a relatively high explanatory rate for biomass allocation trade‐offs (Figure 6). This is consistent with predictions of the trait‐driven theory. Community‐weighted moments of functional traits have influenced the changes in community ecological functions to some extent. In karst climax communities, the CWK of plant functional traits was the primary factor influencing the distribution of community functional traits that affected community biomass, explaining 44.71% of the variation in community biomass. CWK reflects the kurtosis of trait distributions at the community level, with higher kurtosis indicating better adaptation to stable environments (Gross et al. 2017). In karst climax communities, the low kurtosis (CWK) of functional traits suggests that dominant species exhibit divergent trait combinations to adapt to the heterogeneous local environments characteristic of karst ecosystems (Figure S2). Regarding the biomass allocation trade‐off relationship, the CWM of plant functional traits was the primary factor in the distribution of community functional traits affecting the biomass trade‐off allocation of the community, explaining 32.22%–48.21% of the variation in biomass trade‐offs among different forest layers. CWM reflects the functional dominance of a community, emphasizing the role of functional traits of dominant species in the community and supporting the mass ratio hypothesis. The community‐weighted mean of WD, as a major and significant indicator of the community distribution of functional traits that determine RS2 and SS, tends to adopt a resource‐conserving strategy in community biomass allocation. When CWM.WD increases, the biomass of the community decreases, while the proportion of biomass in the renewal layer or succession layer rises, thereby increasing the investment in the growth potential of the community. The CWV characteristics of plant functional traits were the second‐largest factor influencing the biomass allocation trade‐off in the karst climax community. SLA is a key trait linked to carbon use strategies, exhibited reduced carbon accumulation rates in resource‐rich environments when the values are higher. In our study, SLA showed significantly greater community‐weighted variance (CWV) than other functional traits (Figure S2), suggesting that SLA acts as a critical filter governing the assembly of karst forest climax communities. This interpretation was further supported by the multivariate decomposition analysis (Figure 4): across all biomass trade‐off relationships, CWM.SLA maintained its dominant position. Notably, as the allocation to the stable layer biomass increased, both the positive effect of CWM. SLA and the influence of CWV. SLA became more pronounced. These results demonstrate that the distribution of community‐level functional traits fundamentally governs the patterns of biomass allocation. In karst forest communities, the observed biomass trade‐off reflects preferential allocation to the stable layer, with specific leaf area (SLA) emerging as a key trait in the optimization of resource partitioning. In conclusion, in the Maolan Karst climax community, the homogeneity of plant functional traits (CWK) affects the distribution pattern of biomass in the community. The dominance of plant functional traits (represented by CWM) and the diversity of functional traits (represented by CWV) affect the distribution trade‐off of biomass among different forest layers.

### 4.3 Driving Mechanisms of Biomass Trade‐Offs in the Karst Climax Community

As a distinctive non‐zonal vegetation type, karst forests exhibit marked differences in species composition, habitat characteristics, and community structure compared to subtropical evergreen broad‐leaved forests. Our results demonstrate that topographic factors exerted stronger control over aboveground biomass trade‐offs than soil factors (Figure 5). Slope position significantly affected the CWM characteristics of functional traits to promote the allocation of community biomass. The decline in slope position will decrease the trait CWM value, reduce the community's input to the biomass of regeneration and succession layers, and increase the biomass of the stable layers, inhibiting the degree of trade‐offs in the community biomass in RS2 and SS. At the same time, a decrease in the slope position will lead to a decreased CWV values of functional traits, and promote biomass input to the regeneration layer. CWM represents the concentration of ecological strategies of functional traits, and hence can reflect the functional characteristics and advantages and disadvantages of plant groups in a community (Muscarella and Uriarte 2016). CWV demonstrates the variability of plant functional traits and the characteristics of ecological strategies in the community. The variance of traits among strategy species may be significant to rapidly adapting to environmental changes, while the variance of traits in the ecologically conservative species may be less (Guerrero Lizarazo et al. 2021; Kazakou et al. 2022). Our results demonstrate that a lower slope position was associated with reduced CWM.WD and CWM.LTN, indicating a shift toward a resource‐conservative strategy. Concurrently, decreases in CWV.WD, CWV.Max_TH, and CWV.SLA were correlated with enhanced biomass allocation to the renewal layer (Figure S4a). Notably, SLA emerged as a key trait influencing variation in both RS2 and SS trade‐offs (Figure 5a,c), exhibiting particularly high CWV values across communities (Figure 2). The optimal biomass allocation theory posits that plants preferentially allocate biomass to organs acquiring the most limiting resources (Thornley 1972). At the community level, the increased SLA variation enhanced succession layer biomass, whereas higher CWM.SLA boosted biomass in both the renewal and stable layers (Figure S4). These findings demonstrate SLA's pivotal role in governing biomass partitioning within karst forest climax communities.

Therefore, within karst climax communities, slope position serves as a critical environmental filter governing community assembly patterns. The distribution of SLA at the community level significantly regulates trade‐offs in the allocation of biomass, where the community‐weighted mean (CWM.SLA) and variance (CWV.SLA) exhibit differential effects on distinct forest strata.

## 5 Conclusions

Guided by trait‐driven theory, this study explored the mechanism driving the trade‐offs in biomass allocation across forest strata in karst climax communities, highlighting the pivotal role of the community distribution characteristics of plant functional traits. Community‐weighted moments (CWM and CWV) of six functional traits collectively explained 53.91%–71.78% of the variation in biomass allocation, demonstrating their central role in regulating biomass partitioning patterns. Slope position emerged as the dominant topographic factor influencing the distribution of biomass. It mainly influences biomass allocation by regulating the CWM and CWV values of functional traits at the community level. For example, reduced CWM values (CWM.SLA, CWM.LTN, CWM.WD) promoted biomass accumulation in the stable layer, favoring species with higher resource‐use efficiency. Notably, SLA served as a key functional trait, with its CWM and CWV values significantly affecting the patterns of biomass allocation. The broad variation range of SLA underscores its importance in karst community differentiation. While increased CWM.SLA values constrained stable layer biomass allocation, elevated CWV.SLA values counterbalanced this effect through compensatory adjustments in the renewal layer, revealing a dynamic equilibrium between trait mean values and variation.

## Author Contributions

**Dong‐Mei Yuan:** conceptualization (lead), data curation (lead), writing – original draft (lead). **Ling‐Bin Yan:** conceptualization (supporting), writing – review and editing (supporting). **Feng Liu:** methodology (equal). **Hui‐Min Zhang:** data curation (equal). **Xiu‐Gang Cao:** data curation (equal). **Yuan Liu:** writing – review and editing (equal). **Zhi‐Fei Chen:** writing – review and editing (equal). **Li‐Fei Yu:** writing – review and editing (equal).

## Disclosure

Statement on inclusion: Our research is a research result on the plant community at the climax of the subtropical karst zone, based on the analysis of the measured data of the research team. Therefore, with the local dataset available, the public release of detailed data files is not considered for the time being. However, scholars in the same field are very welcome to exchange ideas and provide valuable basic references for their peers' publications.

## Conflicts of Interest

The authors declare no conflicts of interest.

## Supporting information


**Data S1:** Supporting Information.

**Figure S1:** Correlation between abiotic factors.

**Figure S2:** Community distribution characteristics of plant functional traits.

**Figure S3:** PCA analysis of plant functional traits at the community level.

**Figure S4:** Effects of community traits on the biomass of each forest layer.

**Table S1:** Biomass allocation strategy trade‐off value.

**Table S2:** Multivariate decomposition of biomass trade‐offs by biological factors.

**Table S3:** Table of the meanings of abbreviations.

**Table S4:** List of sampled species.

## Data Availability

I confirm that the Data Availability Statement is included in the main file of my submission, and that access to all necessary data files is provided to editors and reviewers. All the required data are uploaded as Supporting Information.
