# Supplementary figures and images for: Community Trait Distributions Drive Biomass Stand Allocation Trade‐Offs in Karst Forests
Source: Ecol Evol. 2026 Feb 8;16(2):e72491. doi: 10.1002/ece3.72491 (PMC12883299; doi:10.1002/ece3.72491)

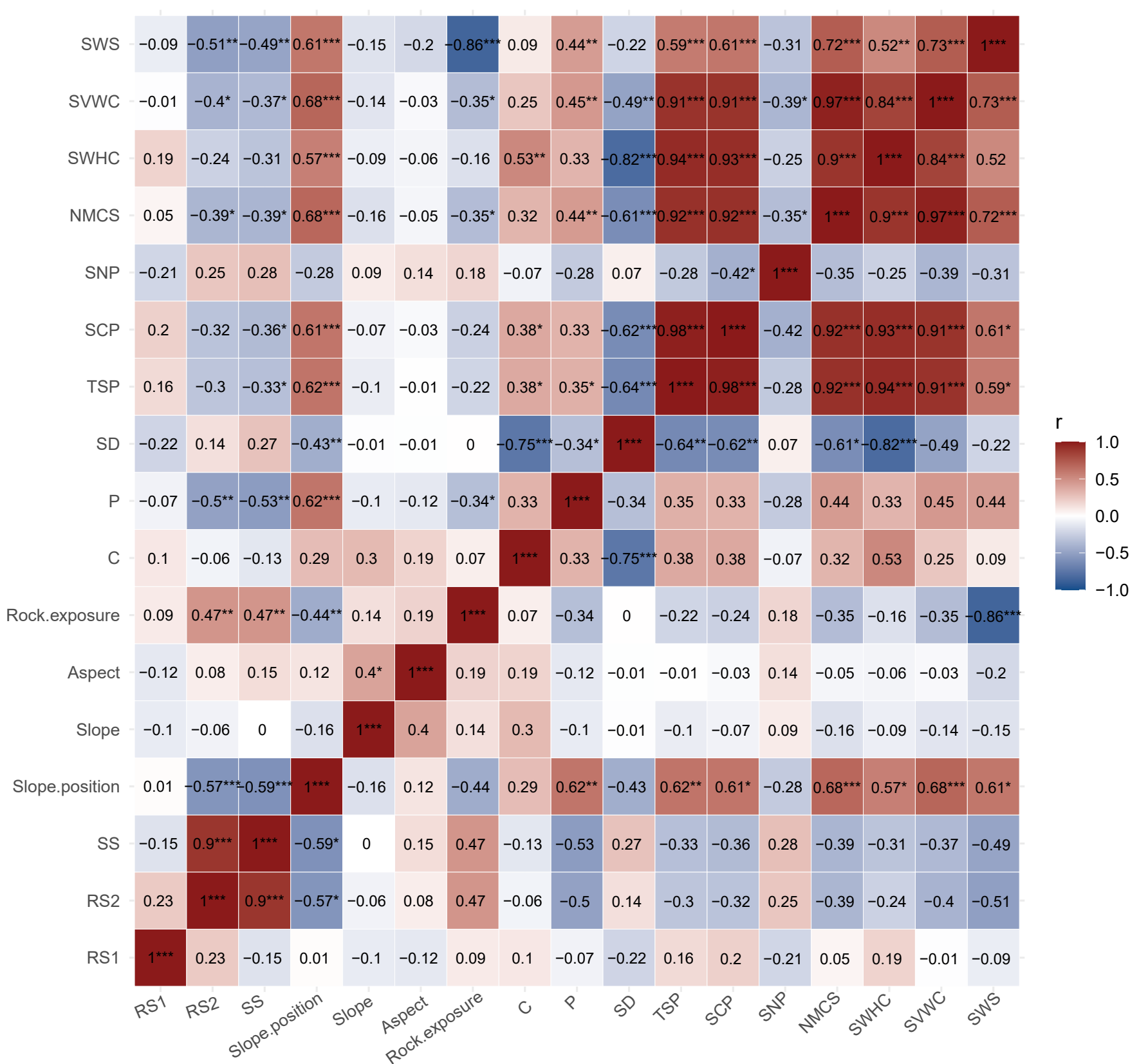

Supplementary Figure 1 Correlation between abiotic factors

Supplement: Supplementary file 2 — Figure S1: Correlation between abiotic factors. [file ECE3-16-e72491-s007.pdf]

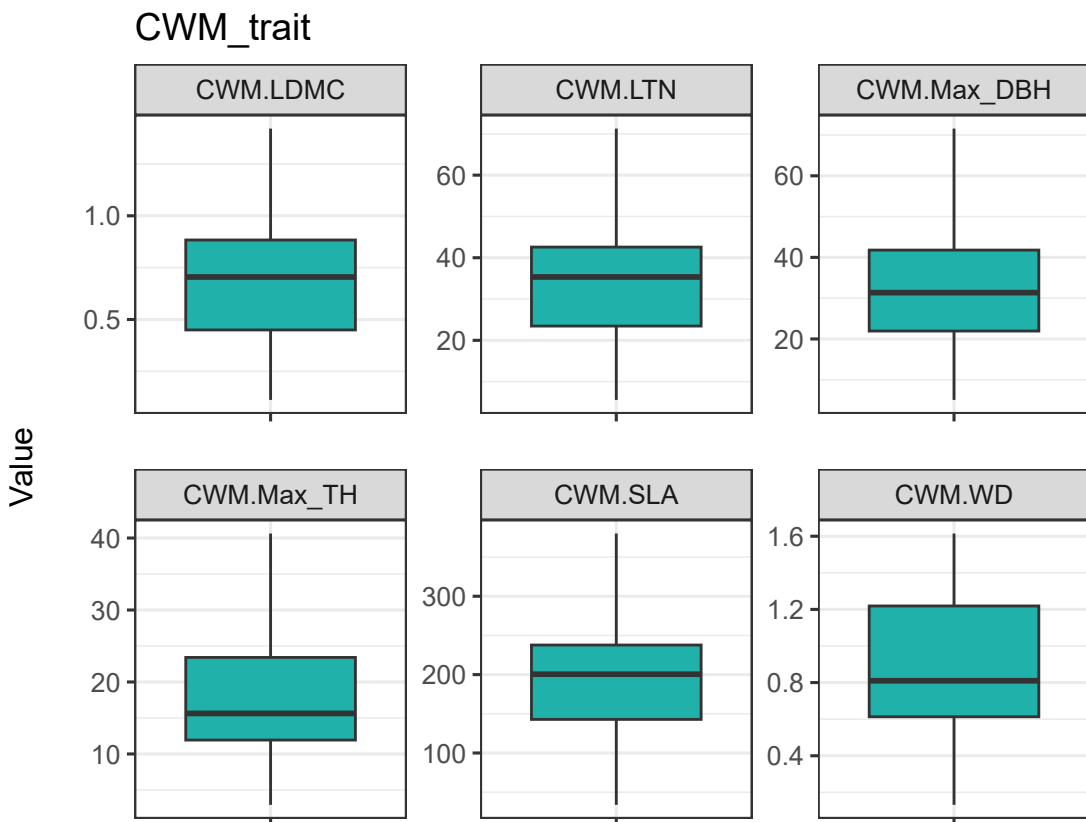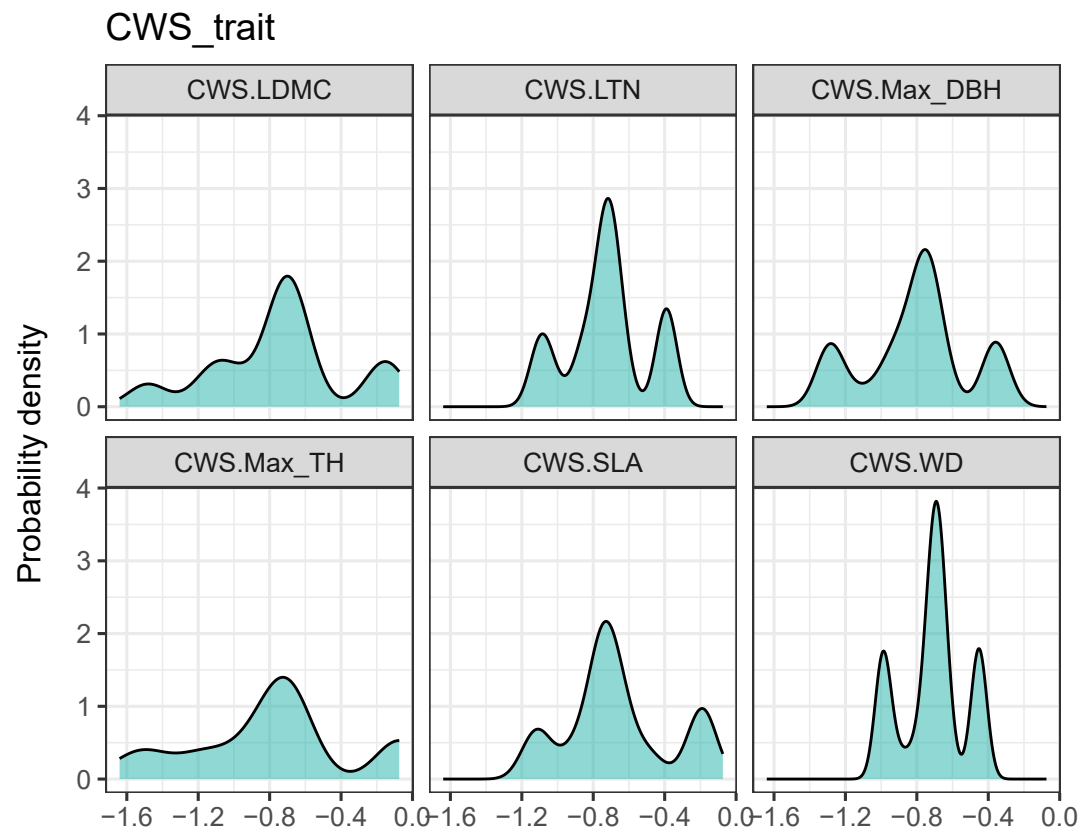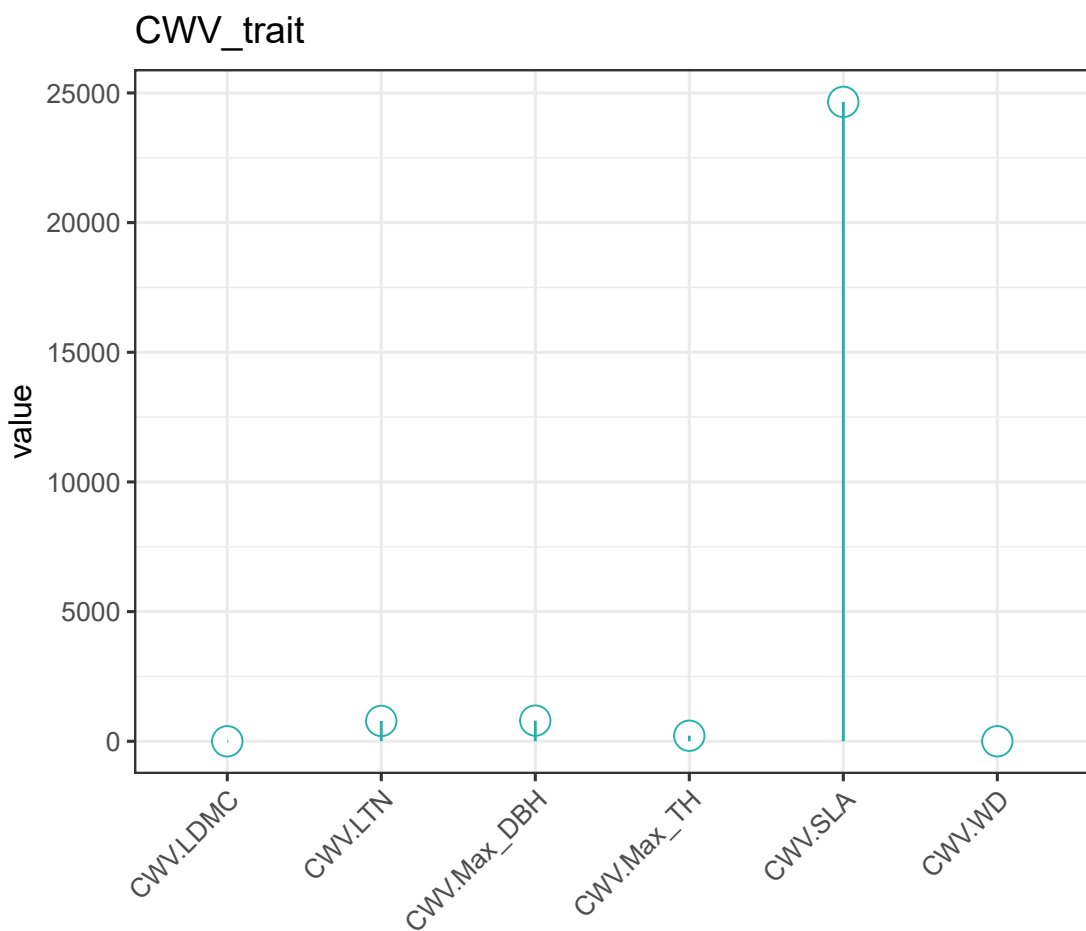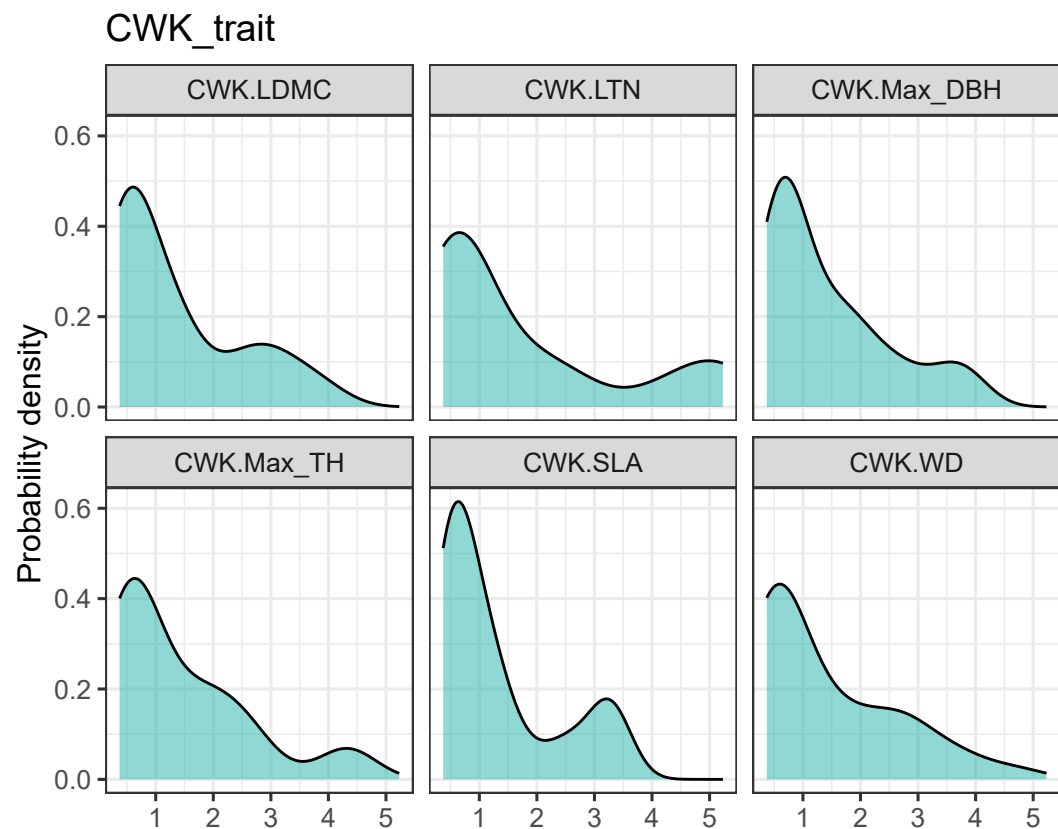

Supplementary Figure 2 Community distribution characteristics of plant functional traits

Supplement: Supplementary file 3 — Figure S2: Community distribution characteristics of plant functional traits. [file ECE3-16-e72491-s008.pdf]

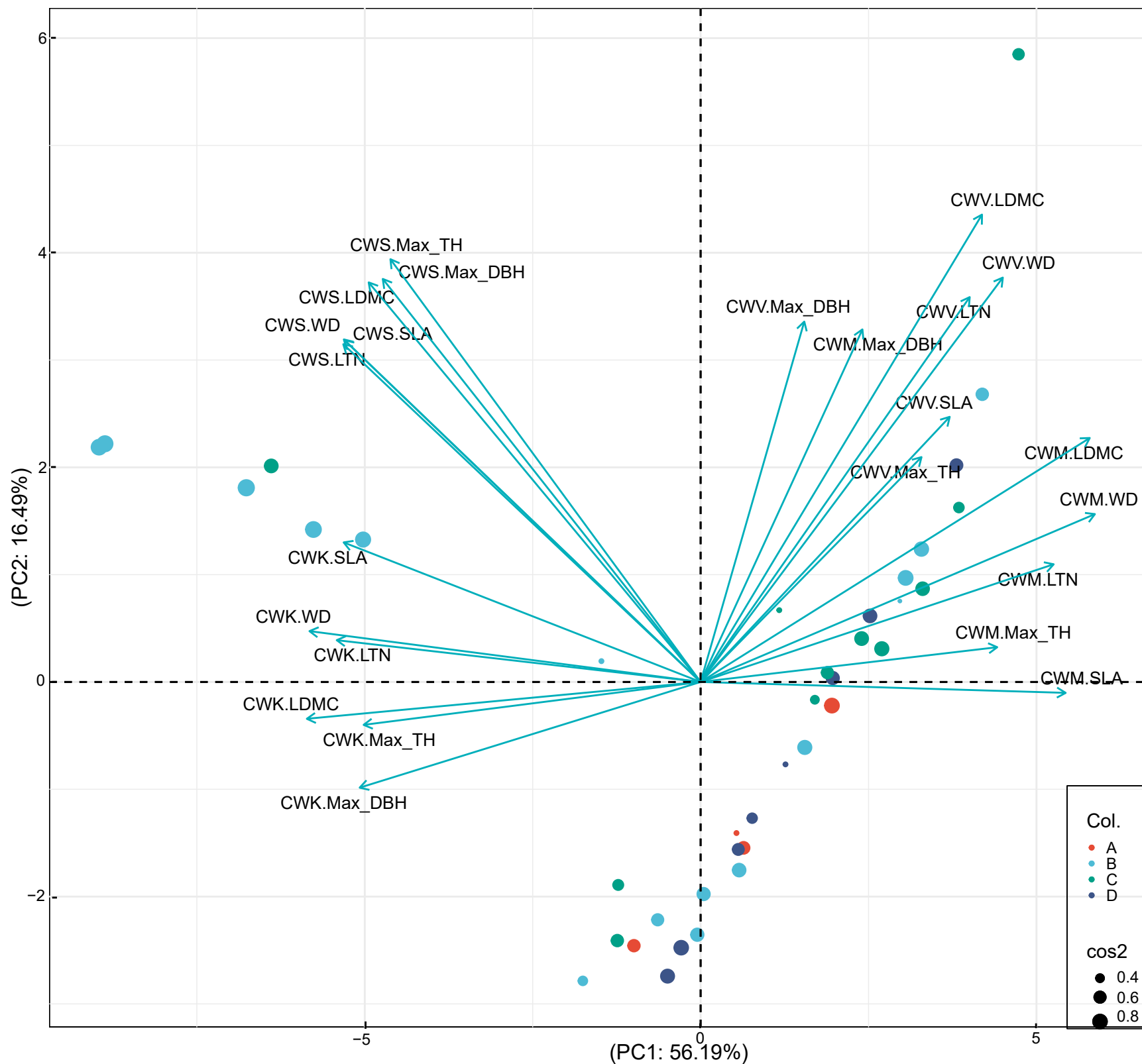

**Supplementary Figure 3 PCA analysis of plant functional traits at the community level**

Supplement: Supplementary file 4 — Figure S3: PCA analysis of plant functional traits at the community level. [file ECE3-16-e72491-s001.pdf]

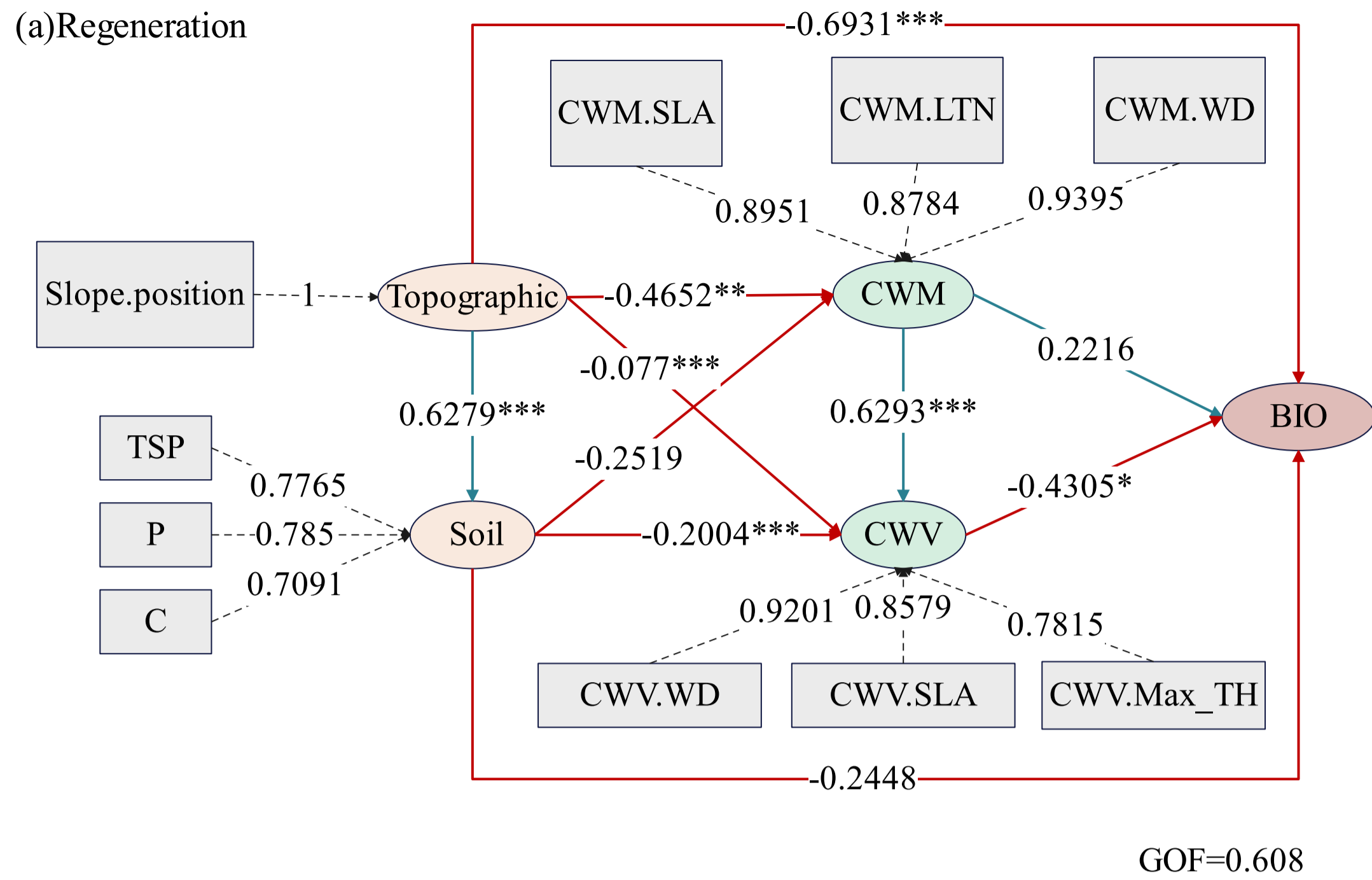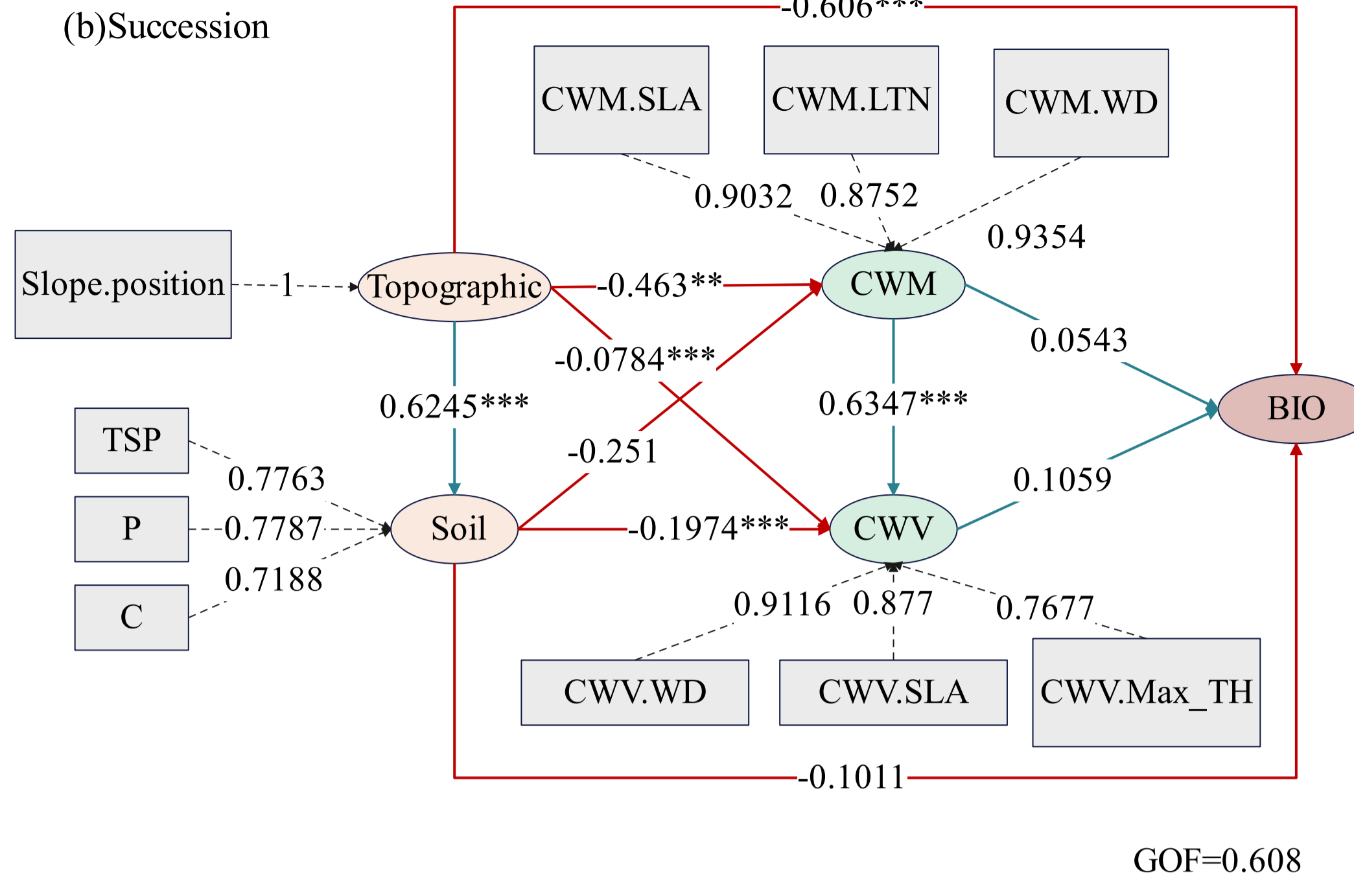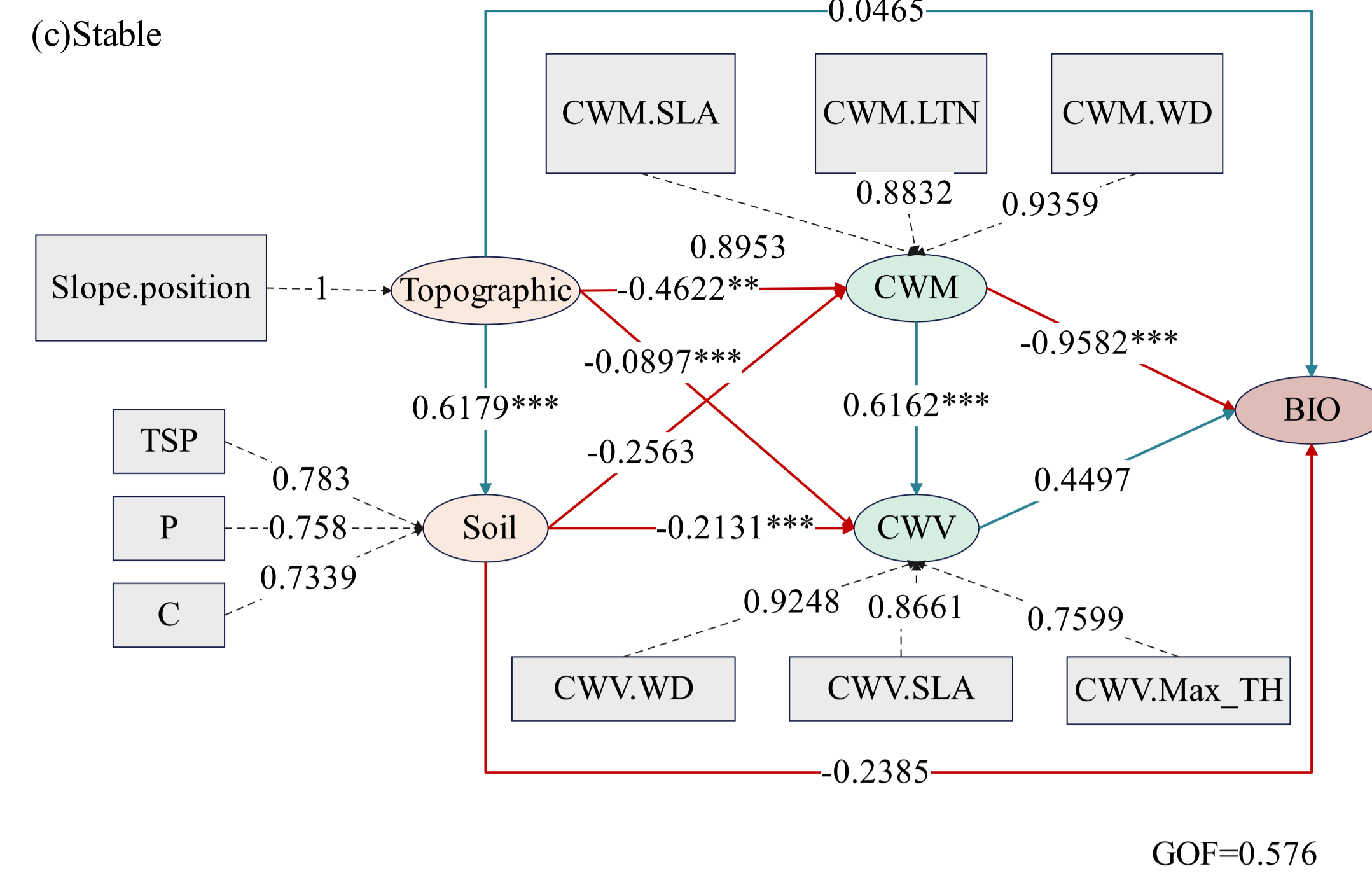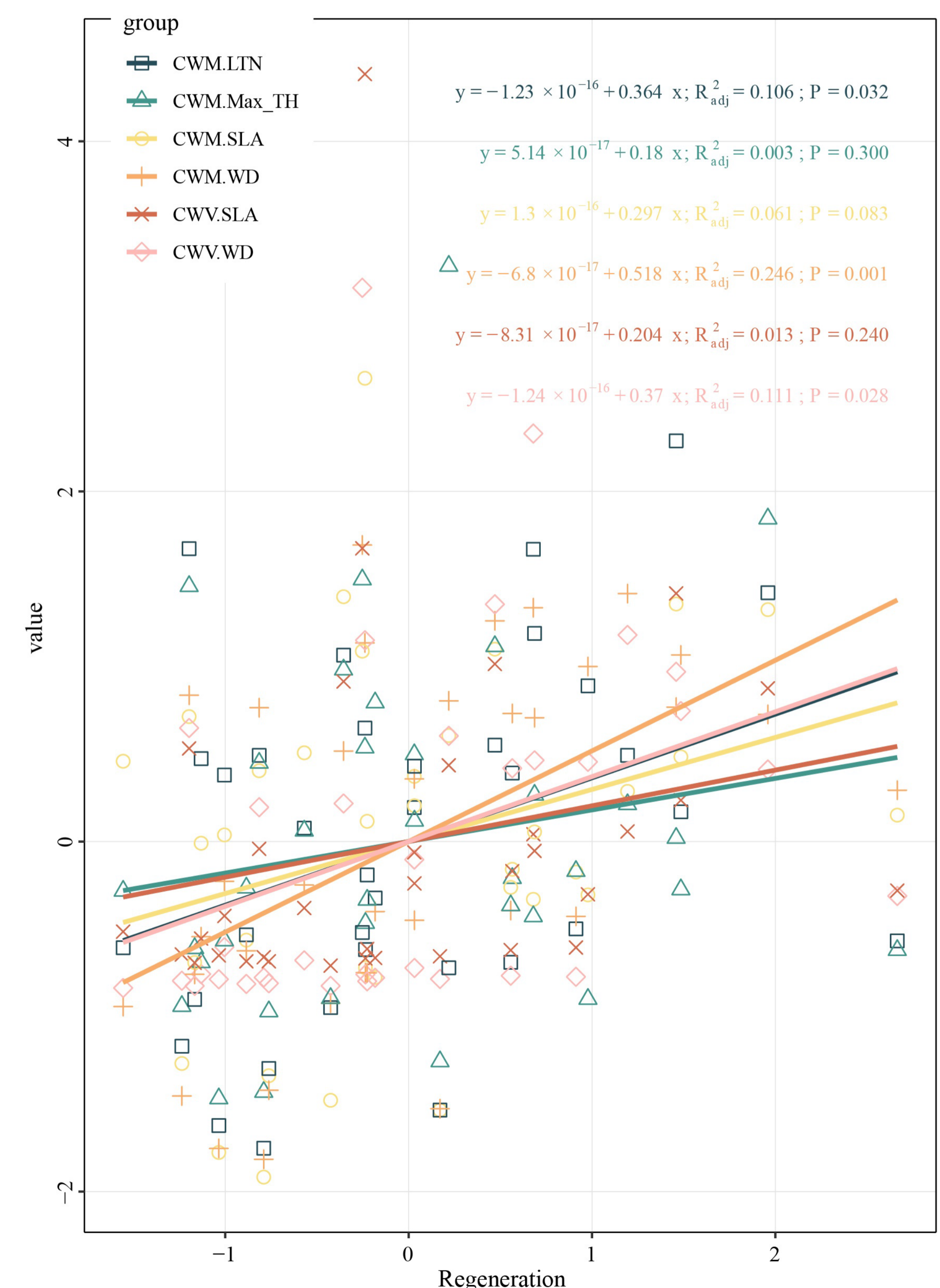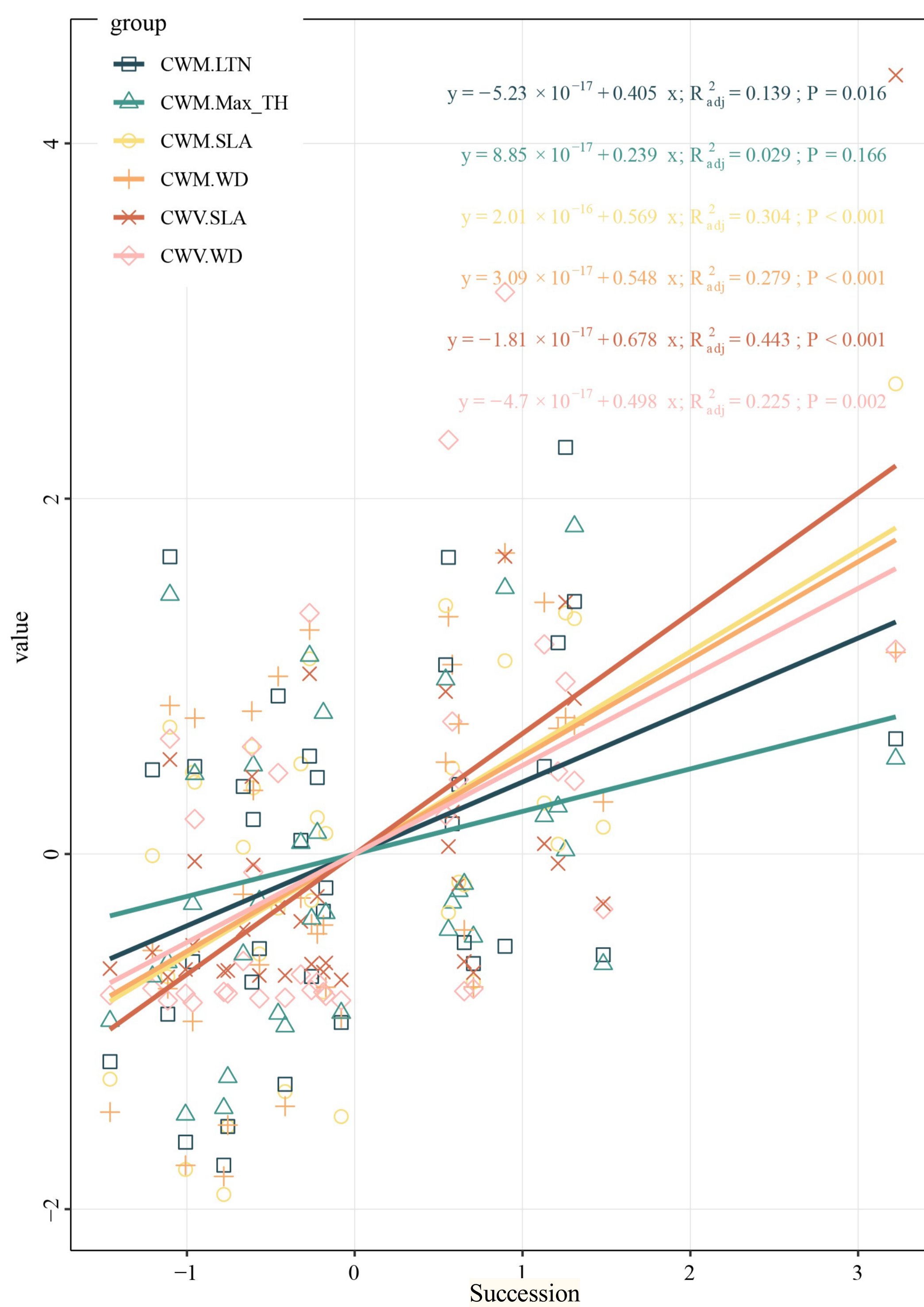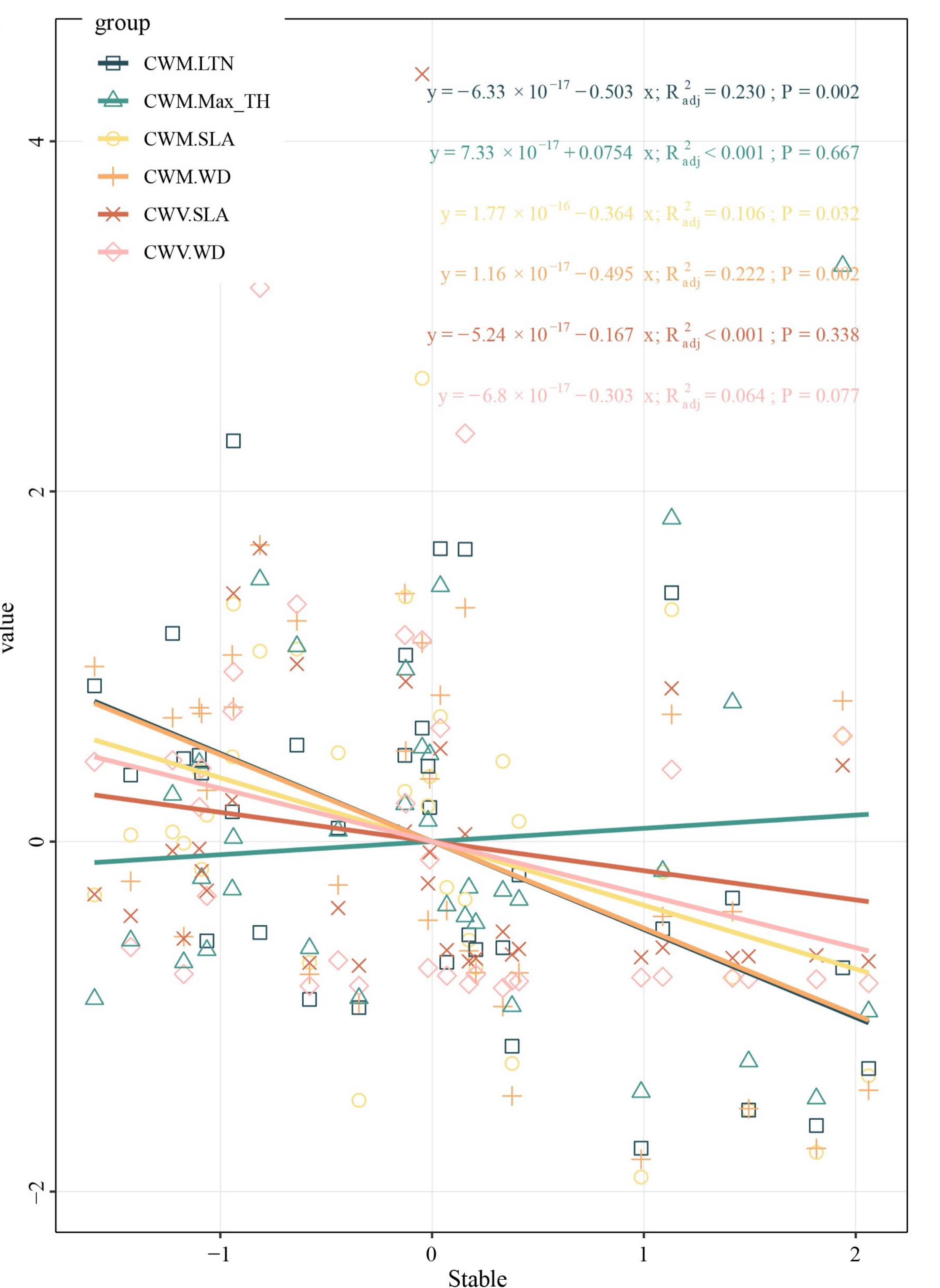

Supplementary Figure 4 Effects of community traits on the biomass of each forest layer

Supplement: Supplementary file 5 — Figure S4: Effects of community traits on the biomass of each forest layer. [file ECE3-16-e72491-s005.pdf]
